# Editorial: Homeostatic Synaptic Plasticity: From Synaptic Circuit Assembly to Neurological Disorders

**DOI:** 10.3389/fncel.2021.695313

**Published:** 2021-05-14

**Authors:** Mathieu Letellier, Lorenzo A. Cingolani

**Affiliations:** ^1^University of Bordeaux, CNRS, Interdisciplinary Institute for Neuroscience, IINS, UMR 5297, Bordeaux, France; ^2^Department of Life Sciences, University of Trieste, Trieste, Italy; ^3^Center for Synaptic Neuroscience and Technology, Istituto Italiano di Tecnologia, Genoa, Italy

**Keywords:** homeostatic synaptic plasticity, neurological disorders, synaptic scaling, extracellular matrix, heterosynaptic plasticity, glia, gene expression

Neuronal networks can be viewed as learning and memory storage devices. They are highly “plastic,” changing the way they process information in response to external stimuli. Yet, they are also highly “tenacious,” with many neuronal networks retaining their functional identity over many years. Hebbian forms of synaptic plasticity, such as long-term potentiation (LTP) and long-term depression (LTD), use positive feedback mechanisms to either reinforce the more active synapses or weaken those that are less active, thus contributing to neuronal networks tuning their outputs to ever-changing external stimuli. By contrast, homeostatic forms of plasticity use negative feedback mechanisms to maintain the overall neuronal output as close as possible to an “internal” prefixed set point, thus restraining neuronal networks from becoming either silent or hyper-excitable. Recent findings have clearly shown that there is not one but multiple forms of homeostatic plasticity occurring at different levels of organization of the brain, from single synapses to dendritic branches to individual neurons to full neuronal networks (Davis, 2013; Nelson and Valakh, 2015; Mullins et al., 2016).

This ebook presents a collection of articles covering molecular and cellular mechanisms that drive forms of homeostatic plasticity whose dysfunction has been proposed to underlie the pathophysiology of many neurological disorders, such as autism spectrum disorder (ASD), schizophrenia, Alzheimer's disease, addiction, intellectual disability, depression and epilepsy (Wondolowski and Dickman, 2013; Fernandes and Carvalho, 2016; Jaudon et al., 2020; Kavalali and Monteggia, 2020). In particular, the Research Topic explores two possibilities to interpret the diseased brain in light of homeostatic plasticity mechanisms. First, neurological disorders could arise because homeostatic plasticity fails to compensate for genetic defects. This can occur either when the genetic mutation directly impairs built-in feedback control systems or when it is so disruptive to overwhelm the buffering capacity of homeostatic plasticity. Second, as it is often the case for epilepsy, ASD or addiction, homeostatic plasticity can become maladaptive. This can occur when deficits at one level of organization of the nervous system (for example impaired synaptic transmission) are compensated for at a different level of organization (for example by heightened cell-wide intrinsic excitability). While such homeostatic compensations can effectively preserve the overall output of a neuronal network, they are also likely to make it unstable or modify how it processes information.

Homeostatic compensations are often presented as relatively slow processes developing in response to prolonged perturbations of neuronal activity and relying on the synthesis of new proteins that regulate key physiological parameters, such as synaptic efficacy, synapse number and membrane excitability. At the transcriptional level, the RE-1 Silencing Transcription factor (REST1) is ideally suited to achieve homeostatic plasticity as it has been shown to repress the expression of various channels and synaptic proteins and has been linked to both homeostatic plasticity and epilepsy. However, the actual role of REST in epilepsy, whether protective or pro-epileptogenic is debated. To clarify the role of REST in epileptogenesis, Carminati et al. have developed a genetic competitive inhibitor to modulate REST activity *in vivo*. The authors demonstrate that inhibiting REST1 reduces the susceptibility to kainate-induced seizures and correlates with an increased expression of REST1 target genes, including potassium channels, GABAergic and glutamatergic receptors. In their perspective article, Lignani et al. further discuss the complex and dynamic functions of REST as well as of one of its targets, HCN1, to better understand the homeostatic adaptations that take place in epilepsy, and why they invariably fail to suppress seizures. The authors propose that a chronic dysregulation of gene expression (the “genetic load”) could transform the contribution of REST and HCN1 from homeostatic to pro-epileptogenic. Accordingly, external genetic interventions (e.g., by enhancing the expression of the potassium channel Kv1.1) may push back neural networks within their physiological boundaries and allow them to take back control of their own homeostasis. Maladaptive homeostatic response is also reported by Yeates and Frank at the *Drosophila* neuromuscular junction (NMJ), where impairment of intracellular calcium gates leads to excessive homeostatic presynaptic depression in response to chronic upregulation of the vesicular glutamate transporter (VGLUT).

Downstream of transcription, Thalhammer et al. discuss the emerging role of activity-dependent alternative splicing as a versatile mechanism to optimize homeostasis. This process not only expands the diversity of isoforms encoded by a single gene but also affects the spatiotemporal dynamics of the corresponding transcripts. The authors provide examples of genes which undergo activity-dependent alternative splicing and whose splice variants exhibit divergent -sometimes opposite- functions in compensating for activity perturbations. Those genes include REST1, the scaffolding protein Homer1 and the P/Q type calcium channels, which regulate intrinsic plasticity, synaptic scaling and presynaptic homeostasis, respectively. More recently, also alternative splicing of BK channels has been shown to participate to homeostatic adaptations by contributing to action potential widening in response to network inactivity (Li et al., 2020). Further downstream along the line of gene expression, protein translation is actively regulated to control homeostatic plasticity in time and space. Dubes et al. review the recent literature addressing the role of microRNAs in various forms of homeostatic plasticity. These non-coding RNAs control the translation of multiple homeostatic effectors including channels, receptors, RNA-binding proteins and cytoskeleton-related proteins. The authors highlight the ability of microRNAs to control homeostasis by repressing their targets either cell-wide or in a compartmentalized fashion (i.e., remotely from the cell body), thus providing autonomy to subcellular functional units such as synapses and dendritic branches.

Whether cell-wide or local, homeostatic plasticity ideally should not compromise information processing occurring at various types of synaptic inputs and outputs. Indeed, most neurons receive synaptic inputs from multiple sources while projecting their axon onto distinct targets, where they form synapses displaying specific functional features. In their study, Goel et al. use the *Drosophila* NMJ to investigate how synapses from an individual neuron homeostatically adapt their strength according to the muscle targets they innervate. The authors identify target-specific homeostatic mechanisms that simultaneously balance for hypo- and hyper-innervation through a differential contribution of pre- and post-synaptic signaling pathways. This study thus highlights the diversity of the homeostatic mechanisms simultaneously implemented by a single neuron to accommodate the requirements of multiple types of outputs. In line with these findings, Lee and Kirkwood review recent evidence showing that neurons embedded in complex sensory networks of the mammalian CNS implement homeostatic synaptic plasticity in an input-specific manner following sensory deprivation. They discuss the role of the “sliding threshold” as a major *in vivo* mechanism to homeostatically adjust the propensity for future LTP and LTD at individual connections depending on prior experience. In contrast, synaptic scaling, in which the efficacy of all synapses is uniformly modified, may occur to stabilize neuronal activity under more extreme activity perturbations, for instance following pharmacological manipulations or widespread seizures.

Whether homeostatic adaptations also take place in more physiological situations (e.g., when a subset of synapses undergo Hebbian plasticity) is an enduring question in the field. While Hebbian plasticity is rapidly implemented, homeostatic plasticity is often viewed as a slow process. Yet, both types of plasticity share common signaling pathways and it remains unclear how homeostatic plasticity can operate without erasing Hebbian plasticity. Galanis and Vlachos propose that Hebbian and homeostatic plasticities coexist at the same synapses, thereby limiting each other. In their model, Hebbian plasticity corresponds to the readjustment of the homeostatic set-point allowing for long-term changes to occur at recruited synapses. In turn, the failure of Hebbian plasticity observed in some physiological or pathological situations may represent enhanced homeostasis. The authors further propose a role for the proteolytic processing of the amyloid precursor protein to set the balance between homeostatic and Hebbian synaptic plasticity. Kruijssen and Wierenga discuss an alternative hypothesis, namely that homeostatic plasticity, rather than affecting directly synaptic strength, modifies the ability of synapses to undergo future LTP, depending not only on their own prior experience (the “sliding threshold” hypothesis discussed by Lee and Kirkwood) but also on that of the nearby synapses. In turn, eliciting LTP at individual synapses triggers compensatory changes at nearby synapses through heterosynaptic signaling. The idea that distinct inputs converging onto the same neuron can balance each other is also proposed by Bannon et al. as a mechanism to prevent the runaway dynamics inherent to Hebbian plasticity. In their review, the authors highlight the possible role of weight-dependent heterosynaptic plasticity in normalizing the excitatory drive to hippocampal inhibitory neurons.

Besides synapse-specific mechanisms, mounting evidence point to both permissive and instructive roles of the extracellular matrix (ECM) and glial cells in homeostatic plasticity. Cingolani et al. discuss how ECM remodeling controls localization and function of various types of metabotropic receptors (for glutamate, dopamine, and serotonin). In turn, metabotropic signaling modulates the extracellular environment, for example, by stimulating extracellular proteases. This synergistic crosstalk stabilizes network activity by regulating both synaptic and intrinsic forms of homeostatic plasticity. In a similar vein, Heir and Stellwagen review how the pro-inflammatory cytokine Tumor necrosis factor alpha (TNFα), which is mainly secreted by glial cells, controls various forms of homeostatic plasticity both *in vitro* and *in vivo* by modulating receptor trafficking. Importantly, both ECM and glial factors are amenable to therapeutic interventions, for example for the control of epileptogenesis (Korotchenko et al., 2014).

Finally, the systematic review by Moulin et al. reports some of the strengths and pitfalls of the research carried out in the field of homeostatic plasticity, focusing on the synaptic scaling literature. In addition to the lack of transparency and details regarding experimental and analysis procedures in some research articles, the authors highlight the underrepresentation of studies using *in vivo* models as well as of those investigating functional interactions with Hebbian plasticity. Like the authors, we believe that such studies should be encouraged in the future.

In summary, this Research Topic provides an overview of recent advances in the field of homeostatic plasticity highlighting the complexity and dynamics of the molecular and cellular mechanisms involved. Perhaps more importantly, most articles presented here not only link homeostatic plasticity to neurological diseases such as epilepsy, neurodegenerative and neuropsychiatric disorders but also provide insights into new avenues for therapeutic intervention.

## Author Contributions

All authors listed have made a substantial, direct and intellectual contribution to the work, and approved it for publication.

## Conflict of Interest

The authors declare that the research was conducted in the absence of any commercial or financial relationships that could be construed as a potential conflict of interest.
